# Improved functional prediction of proteins by learning kernel combinations in multilabel settings

**DOI:** 10.1186/1471-2105-8-S2-S12

**Published:** 2007-05-03

**Authors:** Volker Roth, Bernd Fischer

**Affiliations:** 1 ETH Zurich, Institute of Computational Science, Universität-Str. 6, CH-8092 Zurich, Switzerland

## Abstract

**Background:**

We develop a probabilistic model for combining kernel matrices to predict the function of proteins. It extends previous approaches in that it can handle multiple labels which naturally appear in the context of protein function.

**Results:**

Explicit modeling of multilabels significantly improves the capability of learning protein function from multiple kernels. The performance and the interpretability of the inference model are further improved by simultaneously predicting the subcellular localization of proteins and by combining pairwise classifiers to consistent class membership estimates.

**Conclusion:**

For the purpose of functional prediction of proteins, multilabels provide valuable information that should be included adequately in the training process of classifiers. Learning of functional categories gains from co-prediction of subcellular localization. Pairwise separation rules allow very detailed insights into the relevance of different measurements like sequence, structure, interaction data, or expression data. A preliminary version of the software can be downloaded from .

## Background

The problem of developing machine-learning tools for protein function prediction has gained considerable attention during the last years. From a machine learning perspective, this task exceeds the "standard" settings of learning problems in that a protein can be involved in several different biological processes exhibiting more than one function. This means that the objects we want to classify (i.e. the proteins) might belong to several classes, a setting which is referred to as *multilabel classification*. From a biological perspective, the information carried in these multilabels might be relevant for extracting correlations of functional classes. When it comes to predicting the function of new proteins, it is therefore desirable to develop tools that can explicitly handle such multiple labeled objects.

In this work we present a multilabel version of a nonlinear classifier employing Mercer kernels. Such kernel methods have been successfully applied to a variety of biological data analysis problems. One problem of using kernels, however, is the lacking interpretability of the decision functions. In particular, it is difficult to extract further insights into the nature of a given problem from kernel mappings which represent the data in implicitly defined feature spaces. It has been proposed to address this problem by using *multiple *kernels together with some combination rules, where each of the kernels measures different aspects of the data. Methods for learning sparse kernel combinations have the potential to extract *relevant *measurements for a given task. Moreover, the use of multiple kernels addresses the problem of *data fusion *which is a challenging problem in bioinformatics where data can be represented as strings, graphs, or high dimensional expression profiles. Kernels provide a suitable framework for combining such inhomogeneous data under a common matrix representation.

Existing algorithms for combining kernels recast the problem as a *quadratically constrained quadratic program *(QCQP), [1], as a *semi-infinite linear program *(SILP), [2], or within a *sequential minimization optimization *(SMO) framework, [3]. Methods for selecting kernel parameters have also been introduced in the boosting literature, see e.g. [4] or in the context of Gaussian processes, see e.g. [5]. Our method extends these approaches in two major aspects: due to the generative nature of the underlying classification model, it can learn class correlations induced by multilabeled objects and it can be used as a "building block" in hidden Markov models which allow the inclusion of further categorical information, such as the joint prediction of subcellular localization classes and functional classes. We show that these extensions significantly improve the predictive performance on yeast proteins.

## Methods

Our classifier is based on an extension of the *mixture discriminant analysis *(MDA) framework, which forms a link between Gaussian mixture models and discriminant analysis [6]. The algorithm for solving multilabel classification problems emerges as a special case of this clustering approach. In the following we will briefly outline the algorithm which is composed of the "building blocks" *Gaussian mixture models, discriminant analysis, adaptive ridge penalties *and the proper handling of *multilabels*.

### Learning Gaussian mixtures by LDA

The dataset is assumed to be given as a collection of *n *samples ***x***_*i *_∈ ℝ^*d*^, summarized in the (*n *× *d*) matrix *X*. Consider now a Gaussian mixture model with *K *mixture components which share an identical covariance matrix Σ. Under this model, the data log-likelihood reads

lmix=∑i=1nlog⁡(∑k=1Kπkφ(xi;μk,Σ)),     (1)
 MathType@MTEF@5@5@+=feaafiart1ev1aaatCvAUfKttLearuWrP9MDH5MBPbIqV92AaeXatLxBI9gBaebbnrfifHhDYfgasaacH8akY=wiFfYdH8Gipec8Eeeu0xXdbba9frFj0=OqFfea0dXdd9vqai=hGuQ8kuc9pgc9s8qqaq=dirpe0xb9q8qiLsFr0=vr0=vr0dc8meaabaqaciaacaGaaeqabaqabeGadaaakeaacqWGSbaBdaahaaWcbeqaaiabd2gaTjabdMgaPjabdIha4baakiabg2da9maaqadabaGagiiBaWMaei4Ba8Maei4zaCgaleaacqWGPbqAcqGH9aqpcqaIXaqmaeaacqWGUbGBa0GaeyyeIuoakmaabmaabaWaaabmaeaaiiGacqWFapaCdaWgaaWcbaGaem4AaSgabeaakiab=z8aMjabcIcaOGqadiab+Hha4naaBaaaleaacqWGPbqAaeqaaOGaei4oaSdccmGae0hVd02aaSbaaSqaaiabdUgaRbqabaGccqGGSaalcqqHJoWucqGGPaqkaSqaaiabdUgaRjabg2da9iabigdaXaqaaiabdUealbqdcqGHris5aaGccaGLOaGaayzkaaGaeiilaWIaaCzcaiaaxMaadaqadaqaaiabigdaXaGaayjkaiaawMcaaaaa@5B8A@

where the mixing proportions *π*_*k *_sum to one, and *ϕ *denotes a Gaussian density. The classical EM-algorithm, [7], provides a convenient method for maximizing *l*^*mix*^: in the E-step one computes the assignment probabilities *P*(*C*_*k*_|***x***_*i*_) of objects ***x***_*i *_to classes *C*_*k*_, while in the M-step the current parameters of the Gaussian modes are replaced by the maximum likelihood estimates.

*Linear discriminant analysis *(LDA) is a time-honored classifier that (asymptotically) finds the correct class boundaries if the class-conditional densities are Gaussians with common covariance, which is exactly the supervised version of our model (1). This optimality is due to the fact that for given *class labels*, the maximum likelihood parameters of the model (1) can be found by LDA, see e.g. [8]. This result has been generalized in [6], where it has been shown that in the unsupervised "clustering" case the M-step can be carried out via a *weighted and augmented *LDA: class labels are mimicked by replicating the *n *observations *K *times, with the *k*-th replication having observation weights *P*(*C*_*k*_|***x***_*i*_) and the "class label" *k*.

Following [9], any (standard) LDA problem can be restated as an *optimal scoring *problem. Let the class-memberships of the *n *data vectors be coded as a matrix *Z*, the (*i*, *k*)-th entry of which equals one if the *i*-th observation belongs to class *k*. The point of optimal scoring is to turn categorical variables into quantitative ones: a score vector ***θ ***assigns real numbers to the *K *levels of the categorical response variable, i.e. to the entries in the columns of *Z*. The simultaneous estimation of a sequence of scores ***θ***_*k *_and regression coefficients *β*_*k*_, *k *= 1,...,*K*, constitutes the optimal scoring problem: minimize

∑k=1K||Zθk−Xβk||2     (2)
 MathType@MTEF@5@5@+=feaafiart1ev1aaatCvAUfKttLearuWrP9MDH5MBPbIqV92AaeXatLxBI9gBaebbnrfifHhDYfgasaacH8akY=wiFfYdH8Gipec8Eeeu0xXdbba9frFj0=OqFfea0dXdd9vqai=hGuQ8kuc9pgc9s8qqaq=dirpe0xb9q8qiLsFr0=vr0=vr0dc8meaabaqaciaacaGaaeqabaqabeGadaaakeaadaaeWaqaaiabcYha8jabcYha8jabdQfaAHGadiab=H7aXnaaBaaaleaacqWGRbWAaeqaaOGaeyOeI0IaemiwaGfaleaacqWGRbWAcqGH9aqpcqaIXaqmaeaacqWGlbWsa0GaeyyeIuoakiab=j7aInaaBaaaleaacqWGRbWAaeqaaOGaeiiFaWNaeiiFaW3aaWbaaSqabeaacqaIYaGmaaGccaWLjaGaaCzcamaabmaabaGaeGOmaidacaGLOaGaayzkaaaaaa@47FB@

under the orthogonality constraint Θ^⊤^*Z*^⊤^*Z*Θ = *I*_*K*_/*n*, where Θ = (***θ***_1_,...,***θ***_*K*_) and *I*_*K *_denotes the (*K *× *K*) identity matrix. In [9] an algorithm for this problem has been proposed, whose main ingredient is a multiple linear regression of the scored responses *Z****θ***_*k *_against the data matrix *X*. The algorithm starts with Θ = *I*_*K*_, and the optimal scores are derived from the solution of the multiple regression problem via an eigen-decomposition. It can be shown that solutions to (2) must satisfy a certain orthogonality constraint which allows us to start with a (*K *× *K *- 1) scoring matrix Θ' that is orthogonal to a *K*-vector of ones. In the following we will always consider this latter variant which is beneficial since it reduces the number of regressions to *K *- 1. For simplicity in notation we will still write Θ instead of Θ'.

Returning to the above *weighted and augmented *LDA problem, it has been shown in [6] that the solution for this problem can be found by the standard *optimal scoring *version of LDA after replacing the class indicator matrix *Z *by its "blurred" counterpart Z˜
 MathType@MTEF@5@5@+=feaafiart1ev1aaatCvAUfKttLearuWrP9MDH5MBPbIqV92AaeXatLxBI9gBaebbnrfifHhDYfgasaacH8akY=wiFfYdH8Gipec8Eeeu0xXdbba9frFj0=OqFfea0dXdd9vqai=hGuQ8kuc9pgc9s8qqaq=dirpe0xb9q8qiLsFr0=vr0=vr0dc8meaabaqaciaacaGaaeqabaqabeGadaaakeaacuWGAbGwgaacaaaa@2DF8@. The rows of Z˜
 MathType@MTEF@5@5@+=feaafiart1ev1aaatCvAUfKttLearuWrP9MDH5MBPbIqV92AaeXatLxBI9gBaebbnrfifHhDYfgasaacH8akY=wiFfYdH8Gipec8Eeeu0xXdbba9frFj0=OqFfea0dXdd9vqai=hGuQ8kuc9pgc9s8qqaq=dirpe0xb9q8qiLsFr0=vr0=vr0dc8meaabaqaciaacaGaaeqabaqabeGadaaakeaacuWGAbGwgaacaaaa@2DF8@ consist of the class membership probabilities estimated in the preceding E-step of the EM algorithm.

### Adaptive ridge penalties and kernelization

In order to find sparse solutions, we take a Bayesian viewpoint of the multiple regression problem (2) and specify a prior distribution over the regression coefficients *β*. Following some ideas proposed in the Gaussian process literature, we choose *Automatic Relevance Determination *(ARD) priors, [10], which consist of a product of zero-mean Gaussians with inverse variances *ω*_*i*_:

p(β|ω)∝exp⁡[−∑i=1dωiβi2].     (3)
 MathType@MTEF@5@5@+=feaafiart1ev1aaatCvAUfKttLearuWrP9MDH5MBPbIqV92AaeXatLxBI9gBaebbnrfifHhDYfgasaacH8akY=wiFfYdH8Gipec8Eeeu0xXdbba9frFj0=OqFfea0dXdd9vqai=hGuQ8kuc9pgc9s8qqaq=dirpe0xb9q8qiLsFr0=vr0=vr0dc8meaabaqaciaacaGaaeqabaqabeGadaaakeaacqWGWbaCcqGGOaakiiWacqWFYoGycqGG8baFcqWFjpWDcqGGPaqkcqGHDisTcyGGLbqzcqGG4baEcqGGWbaCdaWadaqaaiabgkHiTmaaqadabaacciGae4xYdC3aaSbaaSqaaiabdMgaPbqabaGccqGFYoGydaqhaaWcbaGaemyAaKgabaGaeGOmaidaaaqaaiabdMgaPjabg2da9iabigdaXaqaaiabdsgaKbqdcqGHris5aaGccaGLBbGaayzxaaGaeiOla4IaaCzcaiaaxMaadaqadaqaaiabiodaZaGaayjkaiaawMcaaaaa@500C@

The hyper-parameters *ω*_*i *_encode the "relevance" of the *i*-th variable in the linear regression. A method called *adaptive ridge regression *finds the hyper-parameters by requiring that the mean prior variance is proportional to 1/*λ*, cf. [11]: 1d∑i=1d1ωi=1λ
 MathType@MTEF@5@5@+=feaafiart1ev1aaatCvAUfKttLearuWrP9MDH5MBPbIqV92AaeXatLxBI9gBaebbnrfifHhDYfgasaacH8akY=wiFfYdH8Gipec8Eeeu0xXdbba9frFj0=OqFfea0dXdd9vqai=hGuQ8kuc9pgc9s8qqaq=dirpe0xb9q8qiLsFr0=vr0=vr0dc8meaabaqaciaacaGaaeqabaqabeGadaaakeaadaWcaaqaaiabigdaXaqaaiabdsgaKbaadaaeWaqaamaalaaabaGaeGymaedabaacciGae8xYdC3aaSbaaSqaaiabdMgaPbqabaaaaOGaeyypa0ZaaSaaaeaacqaIXaqmaeaacqWF7oaBaaaaleaacqWGPbqAcqGH9aqpcqaIXaqmaeaacqWGKbaza0GaeyyeIuoaaaa@3DBB@, *ω*_*i *_> 0, where *λ *is a predefined regularization constant.

The balancing procedure has the effect that some hyper-parameters *ω*_*i *_go to infinity. As a consequence, the coefficients *β*_*i *_are shrinked to zero and the corresponding input variables are discarded. Following [11] it is numerically advantageous to introduce new variables γj,i=ωi/λβj,i
 MathType@MTEF@5@5@+=feaafiart1ev1aaatCvAUfKttLearuWrP9MDH5MBPbIqV92AaeXatLxBI9gBaebbnrfifHhDYfgasaacH8akY=wiFfYdH8Gipec8Eeeu0xXdbba9frFj0=OqFfea0dXdd9vqai=hGuQ8kuc9pgc9s8qqaq=dirpe0xb9q8qiLsFr0=vr0=vr0dc8meaabaqaciaacaGaaeqabaqabeGadaaakeaaiiGacqWFZoWzdaWgaaWcbaGaemOAaOMaeiilaWIaemyAaKgabeaakiabg2da9maakaaabaGae8xYdC3aaSbaaSqaaiabdMgaPbqabaGccqGGVaWlcqWF7oaBaSqabaGccqWFYoGydaWgaaWcbaGaemOAaOMaeiilaWIaemyAaKgabeaaaaa@3EA1@, ci=λ/ωi
 MathType@MTEF@5@5@+=feaafiart1ev1aaatCvAUfKttLearuWrP9MDH5MBPbIqV92AaeXatLxBI9gBaebbnrfifHhDYfgasaacH8akY=wiFfYdH8Gipec8Eeeu0xXdbba9frFj0=OqFfea0dXdd9vqai=hGuQ8kuc9pgc9s8qqaq=dirpe0xb9q8qiLsFr0=vr0=vr0dc8meaabaqaciaacaGaaeqabaqabeGadaaakeaacqWGJbWydaWgaaWcbaGaemyAaKgabeaakiabg2da9maakaaabaacciGae83UdWMaei4la8Iae8xYdC3aaSbaaSqaaiabdMgaPbqabaaabeaaaaa@3692@. Denoting by *D*_***c ***_a diagonal matrix with elements *c*_*i*_, we have to minimize

∑k=1K−1||Z˜θk−XDcγk||2+λγk⊤γks.t.c⊤c=d,ci>0.     (4)
 MathType@MTEF@5@5@+=feaafiart1ev1aaatCvAUfKttLearuWrP9MDH5MBPbIqV92AaeXatLxBI9gBaebbnrfifHhDYfgasaacH8akY=wiFfYdH8Gipec8Eeeu0xXdbba9frFj0=OqFfea0dXdd9vqai=hGuQ8kuc9pgc9s8qqaq=dirpe0xb9q8qiLsFr0=vr0=vr0dc8meaabaqaciaacaGaaeqabaqabeGadaaakeaafaqabeqaeaaaaeaadaaeWaqaaiabcYha8jabcYha8jqbdQfaAzaaiaaccmGae8hUde3aaSbaaSqaaiabdUgaRbqabaGccqGHsislcqWGybawcqWGebardaWgaaWcbaacbmGae43yamgabeaakiab=n7aNnaaBaaaleaacqWGRbWAaeqaaOGaeiiFaWNaeiiFaW3aaWbaaSqabeaacqaIYaGmaaGccqGHRaWkiiGacqqF7oaBcqWFZoWzdaqhaaWcbaGaem4AaSgabaGaeSy==7gaaOGae83SdC2aaSbaaSqaaiabdUgaRbqabaaabaGaem4AaSMaeyypa0JaeGymaedabaGaem4saSKaeyOeI0IaeGymaedaniabggHiLdaakeaaieaacqaFZbWCcqaFUaGlcqaF0baDcqaFUaGlaeaacqGFJbWydaahaaWcbeqaaiabl2==Ubaakiab+ngaJjabg2da9iabdsgaKjabcYcaSaqaaiabdogaJnaaBaaaleaacqWGPbqAaeqaaOGaeyOpa4JaeGimaaJaeiOla4caaiaaxMaacaWLjaWaaeWaaeaacqaI0aanaiaawIcacaGLPaaaaaa@6BDD@

We now consider the case of sharing weights over *J *blocks containing *m *regression coefficients each:

c=(c1,...,c1︸m times,...,cJ,...,cJ)︸m times⊤.     (5)
 MathType@MTEF@5@5@+=feaafiart1ev1aaatCvAUfKttLearuWrP9MDH5MBPbIqV92AaeXatLxBI9gBaebbnrfifHhDYfgasaacH8akY=wiFfYdH8Gipec8Eeeu0xXdbba9frFj0=OqFfea0dXdd9vqai=hGuQ8kuc9pgc9s8qqaq=dirpe0xb9q8qiLsFr0=vr0=vr0dc8meaabaqaciaacaGaaeqabaqabeGadaaakeaaieWacqWFJbWycqGH9aqpdaagaaqaaiabcIcaOiabdogaJnaaBaaaleaacqaIXaqmaeqaaOGaeiilaWIaeiOla4IaeiOla4IaeiOla4IaeiilaWIaem4yam2aaSbaaSqaaiabigdaXaqabaaabaGaemyBa0MaeeiiaaccbaGae4hDaqNae4xAaKMae4xBa0Mae4xzauMae43CamhakiaawIJ=aiabcYcaSiabc6caUiabc6caUiabc6caUiabcYcaSmaayaaabaGaem4yam2aaSbaaSqaaiabdQeakbqabaGccqGGSaalcqGGUaGlcqGGUaGlcqGGUaGlcqGGSaalcqWGJbWydaWgaaWcbaGaemOsaOeabeaakiabcMcaPaWcbaGaemyBa0MaeeiiaaIae4hDaqNae4xAaKMae4xBa0Mae4xzauMae43CamhakiaawIJ=amaaCaaaleqabaGaeSy==7gaaOGaeiOla4IaaCzcaiaaxMaadaqadaqaaiabiwda1aGaayjkaiaawMcaaaaa@6600@

Note that for given weights ***c***, eq. (4) defines a standard ridge-regression problem in the transformed data X˜
 MathType@MTEF@5@5@+=feaafiart1ev1aaatCvAUfKttLearuWrP9MDH5MBPbIqV92AaeXatLxBI9gBaebbnrfifHhDYfgasaacH8akY=wiFfYdH8Gipec8Eeeu0xXdbba9frFj0=OqFfea0dXdd9vqai=hGuQ8kuc9pgc9s8qqaq=dirpe0xb9q8qiLsFr0=vr0=vr0dc8meaabaqaciaacaGaaeqabaqabeGadaaakeaacuWGybawgaacaaaa@2DF4@ = *XD*_***c***_. It is well-known in the kernel literature that the solution vectors γ^
 MathType@MTEF@5@5@+=feaafiart1ev1aaatCvAUfKttLearuWrP9MDH5MBPbIqV92AaeXatLxBI9gBaebbnrfifHhDYfgasaacH8akY=wiFfYdH8Gipec8Eeeu0xXdbba9frFj0=OqFfea0dXdd9vqai=hGuQ8kuc9pgc9s8qqaq=dirpe0xb9q8qiLsFr0=vr0=vr0dc8meaabaqaciaacaGaaeqabaqabeGadaaakeaaiiWacuWFZoWzgaqcaaaa@2E6B@_*k *_lie in the span of these input data, i.e. γ^
 MathType@MTEF@5@5@+=feaafiart1ev1aaatCvAUfKttLearuWrP9MDH5MBPbIqV92AaeXatLxBI9gBaebbnrfifHhDYfgasaacH8akY=wiFfYdH8Gipec8Eeeu0xXdbba9frFj0=OqFfea0dXdd9vqai=hGuQ8kuc9pgc9s8qqaq=dirpe0xb9q8qiLsFr0=vr0=vr0dc8meaabaqaciaacaGaaeqabaqabeGadaaakeaaiiWacuWFZoWzgaqcaaaa@2E6B@_*k *_= X˜
 MathType@MTEF@5@5@+=feaafiart1ev1aaatCvAUfKttLearuWrP9MDH5MBPbIqV92AaeXatLxBI9gBaebbnrfifHhDYfgasaacH8akY=wiFfYdH8Gipec8Eeeu0xXdbba9frFj0=OqFfea0dXdd9vqai=hGuQ8kuc9pgc9s8qqaq=dirpe0xb9q8qiLsFr0=vr0=vr0dc8meaabaqaciaacaGaaeqabaqabeGadaaakeaacuWGybawgaacaaaa@2DF4@^⊤^***α***_*k*_, which means that the data enter the model only in form of the Gram matrix (or Mercer kernel) X˜
 MathType@MTEF@5@5@+=feaafiart1ev1aaatCvAUfKttLearuWrP9MDH5MBPbIqV92AaeXatLxBI9gBaebbnrfifHhDYfgasaacH8akY=wiFfYdH8Gipec8Eeeu0xXdbba9frFj0=OqFfea0dXdd9vqai=hGuQ8kuc9pgc9s8qqaq=dirpe0xb9q8qiLsFr0=vr0=vr0dc8meaabaqaciaacaGaaeqabaqabeGadaaakeaacuWGybawgaacaaaa@2DF4@X˜
 MathType@MTEF@5@5@+=feaafiart1ev1aaatCvAUfKttLearuWrP9MDH5MBPbIqV92AaeXatLxBI9gBaebbnrfifHhDYfgasaacH8akY=wiFfYdH8Gipec8Eeeu0xXdbba9frFj0=OqFfea0dXdd9vqai=hGuQ8kuc9pgc9s8qqaq=dirpe0xb9q8qiLsFr0=vr0=vr0dc8meaabaqaciaacaGaaeqabaqabeGadaaakeaacuWGybawgaacaaaa@2DF4@^⊤^. Since we have assumed that a weight *c*_*i *_is shared over a whole block of *m *features, we can decompose this kernel as a weighted sum of *J *individual kernels:

K:=X˜X˜⊤=∑j=1Jcj2X˜(j)X˜(j)⊤=:∑j=1Jcj2Kj.     (6)
 MathType@MTEF@5@5@+=feaafiart1ev1aaatCvAUfKttLearuWrP9MDH5MBPbIqV92AaeXatLxBI9gBaebbnrfifHhDYfgasaacH8akY=wiFfYdH8Gipec8Eeeu0xXdbba9frFj0=OqFfea0dXdd9vqai=hGuQ8kuc9pgc9s8qqaq=dirpe0xb9q8qiLsFr0=vr0=vr0dc8meaabaqaciaacaGaaeqabaqabeGadaaakeaacqWGlbWscqGG6aGocqGH9aqpcuWGybawgaacaiqbdIfayzaaiaWaaWbaaSqabeaacqWI9=VBaaGccqGH9aqpdaaeWaqaaiabdogaJnaaDaaaleaacqWGQbGAaeaacqaIYaGmaaGccuWGybawgaacamaaBaaaleaacqGGOaakcqWGQbGAcqGGPaqkaeqaaOGafmiwaGLbaGaadaqhaaWcbaGaeiikaGIaemOAaOMaeiykaKcabaGaeSy==7gaaaqaaiabdQgaQjabg2da9iabigdaXaqaaiabdQeakbqdcqGHris5aOGaeyypa0JaeiOoaOZaaabmaeaacqWGJbWydaqhaaWcbaGaemOAaOgabaGaeGOmaidaaOGaem4saS0aaSbaaSqaaiabdQgaQbqabaaabaGaemOAaOMaeyypa0JaeGymaedabaGaemOsaOeaniabggHiLdGccqGGUaGlcaWLjaGaaCzcamaabmaabaGaeGOnaydacaGLOaGaayzkaaaaaa@6082@

with X˜
 MathType@MTEF@5@5@+=feaafiart1ev1aaatCvAUfKttLearuWrP9MDH5MBPbIqV92AaeXatLxBI9gBaebbnrfifHhDYfgasaacH8akY=wiFfYdH8Gipec8Eeeu0xXdbba9frFj0=OqFfea0dXdd9vqai=hGuQ8kuc9pgc9s8qqaq=dirpe0xb9q8qiLsFr0=vr0=vr0dc8meaabaqaciaacaGaaeqabaqabeGadaaakeaacuWGybawgaacaaaa@2DF4@_(*j*) _denoting a (*n *× *m*) sub-matrix of X˜
 MathType@MTEF@5@5@+=feaafiart1ev1aaatCvAUfKttLearuWrP9MDH5MBPbIqV92AaeXatLxBI9gBaebbnrfifHhDYfgasaacH8akY=wiFfYdH8Gipec8Eeeu0xXdbba9frFj0=OqFfea0dXdd9vqai=hGuQ8kuc9pgc9s8qqaq=dirpe0xb9q8qiLsFr0=vr0=vr0dc8meaabaqaciaacaGaaeqabaqabeGadaaakeaacuWGybawgaacaaaa@2DF4@ consisting of one block of *m *input features. With the above expression we have arrived at the desired framework for learning sparse combinations of kernel matrices: the kernel matrices *K*_*j *_in (6) which have been formally introduced by partitioning an initial feature set into *J *feature blocks can be substituted by arbitrary kernels fulfilling the positive-semidefiniteness condition of valid dot product matrices. On the technical side, we have to minimize the "kernelized" version of eq. (4)

∑k=1K−1||Z˜θk−(∑j=1Jcj2Kj)αk||2+λαk⊤(∑j=1Jcj2Kj)αk     (7)
 MathType@MTEF@5@5@+=feaafiart1ev1aaatCvAUfKttLearuWrP9MDH5MBPbIqV92AaeXatLxBI9gBaebbnrfifHhDYfgasaacH8akY=wiFfYdH8Gipec8Eeeu0xXdbba9frFj0=OqFfea0dXdd9vqai=hGuQ8kuc9pgc9s8qqaq=dirpe0xb9q8qiLsFr0=vr0=vr0dc8meaabaqaciaacaGaaeqabaqabeGadaaakeaadaaeWaqaaiabcYha8jabcYha8jqbdQfaAzaaiaaccmGae8hUde3aaSbaaSqaaiabdUgaRbqabaGccqGHsislcqGGOaakdaaeWaqaaiabdogaJnaaDaaaleaacqWGQbGAaeaacqaIYaGmaaGccqWGlbWsdaWgaaWcbaGaemOAaOgabeaaaeaacqWGQbGAcqGH9aqpcqaIXaqmaeaacqWGkbGsa0GaeyyeIuoakiabcMcaPiab=f7aHnaaBaaaleaacqWGRbWAaeqaaOGaeiiFaWNaeiiFaW3aaWbaaSqabeaacqaIYaGmaaGccqGHRaWkiiGacqGF7oaBcqWFXoqydaqhaaWcbaGaem4AaSgabaGaeSy==7gaaOGaeiikaGYaaabmaeaacqWGJbWydaqhaaWcbaGaemOAaOgabaGaeGOmaidaaOGaem4saS0aaSbaaSqaaiabdQgaQbqabaaabaGaemOAaOMaeyypa0JaeGymaedabaGaemOsaOeaniabggHiLdGccqGGPaqkcqWFXoqydaWgaaWcbaGaem4AaSgabeaakiaaxMaacaWLjaWaaeWaaeaacqaI3aWnaiaawIcacaGLPaaaaSqaaiabdUgaRjabg2da9iabigdaXaqaaiabdUealjabgkHiTiabigdaXaqdcqGHris5aaaa@71E2@

subject to c⊤c=∑j=1Jcj2=d
 MathType@MTEF@5@5@+=feaafiart1ev1aaatCvAUfKttLearuWrP9MDH5MBPbIqV92AaeXatLxBI9gBaebbnrfifHhDYfgasaacH8akY=wiFfYdH8Gipec8Eeeu0xXdbba9frFj0=OqFfea0dXdd9vqai=hGuQ8kuc9pgc9s8qqaq=dirpe0xb9q8qiLsFr0=vr0=vr0dc8meaabaqaciaacaGaaeqabaqabeGadaaakeaaieWacqWFJbWydaahaaWcbeqaaiabl2==Ubaakiab=ngaJjabg2da9maaqadabaGaem4yam2aa0baaSqaaiabdQgaQbqaaiabikdaYaaakiabg2da9iabdsgaKbWcbaGaemOAaOMaeyypa0JaeGymaedabaGaemOsaOeaniabggHiLdaaaa@4024@, *c*_*i *_> 0. The minimizing vectors α^
 MathType@MTEF@5@5@+=feaafiart1ev1aaatCvAUfKttLearuWrP9MDH5MBPbIqV92AaeXatLxBI9gBaebbnrfifHhDYfgasaacH8akY=wiFfYdH8Gipec8Eeeu0xXdbba9frFj0=OqFfea0dXdd9vqai=hGuQ8kuc9pgc9s8qqaq=dirpe0xb9q8qiLsFr0=vr0=vr0dc8meaabaqaciaacaGaaeqabaqabeGadaaakeaaiiGacuWFXoqygaqcaaaa@2E62@_*k*_, *k *= 1,...,*K *- 1 can be found simultaneously in a very efficient way by employing *block conjugate gradient methods *[12]. The optimal weights ***c ***are found iteratively by a fixed-point algorithm similar to that proposed in [11]:

(cj2)new=J∑k=1K−1cj2α^k⊤Kjα^k∑k=1K−1∑l=1Jcl2α^k⊤Klα^k.     (8)
 MathType@MTEF@5@5@+=feaafiart1ev1aaatCvAUfKttLearuWrP9MDH5MBPbIqV92AaeXatLxBI9gBaebbnrfifHhDYfgasaacH8akY=wiFfYdH8Gipec8Eeeu0xXdbba9frFj0=OqFfea0dXdd9vqai=hGuQ8kuc9pgc9s8qqaq=dirpe0xb9q8qiLsFr0=vr0=vr0dc8meaabaqaciaacaGaaeqabaqabeGadaaakeaacqGGOaakcqWGJbWydaqhaaWcbaGaemOAaOgabaGaeGOmaidaaOGaeiykaKYaaSbaaSqaaGqaaiab=5gaUjab=vgaLjab=Dha3bqabaGccqGH9aqpcqWGkbGsdaWcaaqaamaaqadabaGaem4yam2aa0baaSqaaiabdQgaQbqaaiabikdaYaaaiiWakiqb+f7aHzaajaWaa0baaSqaaiabdUgaRbqaaiabl2==UbaaaeaacqWGRbWAcqGH9aqpcqaIXaqmaeaacqWGlbWscqGHsislcqaIXaqma0GaeyyeIuoakiabdUealnaaBaaaleaacqWGQbGAaeqaaOGaf4xSdeMbaKaadaWgaaWcbaGaem4AaSgabeaaaOqaamaaqadabaWaaabmaeaacqWGJbWydaqhaaWcbaGaemiBaWgabaGaeGOmaidaaOGaf4xSdeMbaKaadaqhaaWcbaGaem4AaSgabaGaeSy==7gaaOGaem4saS0aaSbaaSqaaiabdYgaSbqabaGccuGFXoqygaqcamaaBaaaleaacqWGRbWAaeqaaaqaaiabdYgaSjabg2da9iabigdaXaqaaiabdQeakbqdcqGHris5aaWcbaGaem4AaSMaeyypa0JaeGymaedabaGaem4saSKaeyOeI0IaeGymaedaniabggHiLdaaaOGaeiOla4IaaCzcaiaaxMaadaqadaqaaiabiIda4aGaayjkaiaawMcaaaaa@7490@

Practically, if during the iterations a component *c*_*j *_becomes small compared to a predefined accuracy constant, *c*_*j *_is set to zero, and in all regression problems the *j*-th kernel vanishes.

The algorithm proceeds with iterated computations of kernel weights ***c ***and the expansion coefficients ***α***_*k*_, *k *= 1...*K *- 1. We initialize the model with *c*_*j *_= 1 ∀*j*. In our experiments, the initialization did not critically influence the final result, as long as the initial *c*_*j*_'s are non-zero and *J *<*n*. Theoretical uniqueness results, however, are difficult to derive. For the special case without weight sharing (i.e. *J *= *d*) the above method is equivalent to the *LASSO *model of ℓ_1_-penalized regression (see [11]) for which a unique solution always exists if the dimensionality does not exceed the number of samples, *d *≤ *n*. If *d *exceeds *n *(which might be the case for the kernel models considered here), there might exist different solutions which, however, share the same globally optimal value of the functional (4). The experimentally observed insensitivity to different initializations is probably due to the weights-sharing constraints that shrink the number of different *c*_*j*_'s from *d *to *J*. A theoretical analysis of the uniqueness of solutions, however, will be subject of future work.

### Multilabel classification

In multilabel classification problems, an object ***x***_*i *_can belong to more than one class, i.e. it might come with a set of labels *Y*_*i*_. We treat these multilabels in a probabilistic way by assigning to each observation a set of class-membership probabilities. These probabilities might be given explicitly by the supervisor. If such information is not available, they might be estimated uniformly as 1/|*Y*_*i*_| for classes included in the label set *Y*_*i*_, and zero otherwise. After encoding these probabilities in the "blurred" response matrix Z˜
 MathType@MTEF@5@5@+=feaafiart1ev1aaatCvAUfKttLearuWrP9MDH5MBPbIqV92AaeXatLxBI9gBaebbnrfifHhDYfgasaacH8akY=wiFfYdH8Gipec8Eeeu0xXdbba9frFj0=OqFfea0dXdd9vqai=hGuQ8kuc9pgc9s8qqaq=dirpe0xb9q8qiLsFr0=vr0=vr0dc8meaabaqaciaacaGaaeqabaqabeGadaaakeaacuWGAbGwgaacaaaa@2DF8@ (which corresponds to a single E-step in the EM algorithm), we run one optimization step (i.e. M-step) described above.

Kernel discriminant analysis is a generative classifier which implicitly models the classes as Gaussians in the kernel feature space. The effect of multilabels on the classifier during the training phase can be understood intuitively as follows. If there are many objects in the training set which belong to both the classes *C*_*i *_and *C*_*j*_, the respective class centroids ***μ***_*i*_, ***μ***_*j *_will be shrunken towards the averaged value 1/2·(***μ***_*i *_+ ***μ***_*j*_). In this way, the classifier can learn the correlation of class labels and favor the co-prediction of class *i *and *j*.

For discriminant analysis it is straightforward to compute for each object a vector of assignment probabilities to the individual classes *C*_*k*_, see e.g. [9]. In a traditional two-class scenario we would typically assign an object to class *C*_1 _if the corresponding membership probability exceeds 1/2(for equal class priors). In multilabel scenarios, however, an object can belong to different classes so that we have to find a suitable way of thresholding the output probabilities. In analogy to the classical two-class case, we propose to sort the assignment probabilities in decreasing order and assign an object ***x***_*i *_to the first *k *classes in this order such that

**Figure 1:**
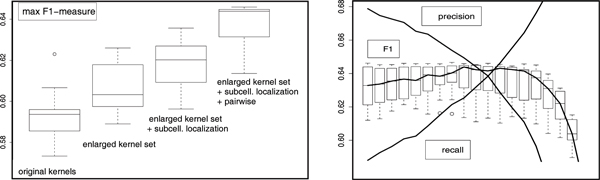
**Performance of different classifiers**. Performance of different classifier variants under cross-validation. Left panel: boxplots of maximum F1 measure. Left to right: 8 original kernels from [1], extended kernel set, combined subcellular location and functional prediction, pairwise learning of the kernel weights (in the combined model). Right panel: precision, recall and F1 for the pairwise classifier used as "building block" in the HMM under variation of the threshold *τ *which controls the label multiplicity.

∑j=1kpsorted(Cj|xi)≥τ,     (9)
 MathType@MTEF@5@5@+=feaafiart1ev1aaatCvAUfKttLearuWrP9MDH5MBPbIqV92AaeXatLxBI9gBaebbnrfifHhDYfgasaacH8akY=wiFfYdH8Gipec8Eeeu0xXdbba9frFj0=OqFfea0dXdd9vqai=hGuQ8kuc9pgc9s8qqaq=dirpe0xb9q8qiLsFr0=vr0=vr0dc8meaabaqaciaacaGaaeqabaqabeGadaaakeaadaaeWaqaaiabdchaWnaaCaaaleqabaacbaGae83CamNae83Ba8Mae8NCaiNae8hDaqNae8xzauMae8hzaqgaaOGaeiikaGIaem4qam0aaSbaaSqaaiabdQgaQbqabaGccqGG8baFieWacqGF4baEdaWgaaWcbaGaemyAaKgabeaakiabcMcaPiabgwMiZIGaciab9r8a0jabcYcaSiaaxMaacaWLjaWaaeWaaeaacqaI5aqoaiaawIcacaGLPaaaaSqaaiabdQgaQjabg2da9iabigdaXaqaaiabdUgaRbqdcqGHris5aaaa@4E6B@

where *τ *is a predefined threshold (e.g. *τ *= 1/2). By varying *τ *one can record the usual precision-recall curves, cf. Figure 1 for an example.

**Algorithm 1 **Multilabel kernel learning via AdR regression

**Training**: /* we start with one E-step */

compute "blurred" (*n *× *K*) response matrix Z˜
 MathType@MTEF@5@5@+=feaafiart1ev1aaatCvAUfKttLearuWrP9MDH5MBPbIqV92AaeXatLxBI9gBaebbnrfifHhDYfgasaacH8akY=wiFfYdH8Gipec8Eeeu0xXdbba9frFj0=OqFfea0dXdd9vqai=hGuQ8kuc9pgc9s8qqaq=dirpe0xb9q8qiLsFr0=vr0=vr0dc8meaabaqaciaacaGaaeqabaqabeGadaaakeaacuWGAbGwgaacaaaa@2DF8@ encoding the membership probabilities.

/* now follows one single M-step */

compute initial (*n *× *K *- 1) scoring matrix Θ_0_, see [9] for details;

Initialize *c*_*i *_= 1, ∀ *i *= 1,...,*J*;

repeat

compute ***α***_*k*_, *k *= 1,...,*K *- 1 as the solution of the linear systems (7);

recompute the kernel weights ***c***^(*t*+1)^, see (8);

**until **||***c***^(*t*+1) ^- ***c***^(*t*)^|| <*ε*

compute fitted values and projection matrix for the discriminant analysis subspace, see [9,13];

**Prediction**:

compute projected test object x˜
 MathType@MTEF@5@5@+=feaafiart1ev1aaatCvAUfKttLearuWrP9MDH5MBPbIqV92AaeXatLxBI9gBaebbnrfifHhDYfgasaacH8akY=wiFfYdH8Gipec8Eeeu0xXdbba9frFj0=OqFfea0dXdd9vqai=hGuQ8kuc9pgc9s8qqaq=dirpe0xb9q8qiLsFr0=vr0=vr0dc8meaabaqaciaacaGaaeqabaqabeGadaaakeaaieWacuWF4baEgaacaaaa@2E3C@_* _and Mahalanobis distances to class centroids;

compute class membership probabilities *P*(*C*_*k*_|x_*_) and extract multilabels according to eq.(9).

### Model selection

For the purpose of model selection, we assume that we are given a set of kernel matrices over multi-labeled objects. We further assume that the rule of deriving "fractional labels" from the multilabels is given. In the absence of further prior knowledge (which is probably the case in most real-world applications), we assume that the fractional labels are derived by averaging over all classes to which an object is assigned. Under these assumptions, the model contains only two free parameters, namely the regularization constant *λ *in eq. (7) and the threshold *τ *for predicting multilabels in eq. (9). In our experiments, the former is estimated via cross-validation on the training sets. A unique multilabel-threshold can also be estimated by cross-validation, but in this work we report the full precision-recall-F1-curves obtained by varying this threshold, see Figure 1.

### Functional classes and subcellular localization

In several classification tasks in bioinformatics, more than one classification scheme is available to assign the data to certain groups. Proteins, for instance, can be classified not only according to their *function*, but also according to their *subcellular localization*. For the yeast genome, such a localization-based scheme is available from CYGD, see Table 2. If for some proteins their subcellular localization can be predicted with high reliability, and if we find high correlations between the corresponding localization classes and certain functional classes, we can potentially exploit this prior knowledge to increase the performance of the functional classification.

**Figure 2:**
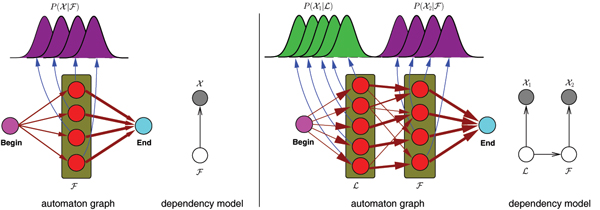
**Graphical representation of hidden Markov models**. Graphical representation of *hidden Markov models*. Left: standard 4-class discriminant analysis with 4 Gaussian emission probabilities. ℱ
 MathType@MTEF@5@5@+=feaafiart1ev1aaatCvAUfKttLearuWrP9MDH5MBPbIqV92AaeXatLxBI9gBamrtHrhAL1wy0L2yHvtyaeHbnfgDOvwBHrxAJfwnaebbnrfifHhDYfgasaacH8akY=wiFfYdH8Gipec8Eeeu0xXdbba9frFj0=OqFfea0dXdd9vqai=hGuQ8kuc9pgc9s8qqaq=dirpe0xb9q8qiLsFr0=vr0=vr0dc8meaabaqaciaacaGaaeqabaWaaeGaeaaakeaaimaacqWFXeIraaa@3787@ denotes the hidden random variable "functional class", X
 MathType@MTEF@5@5@+=feaafiart1ev1aaatCvAUfKttLearuWrP9MDH5MBPbIqV92AaeXatLxBI9gBamrtHrhAL1wy0L2yHvtyaeHbnfgDOvwBHrxAJfwnaebbnrfifHhDYfgasaacH8akY=wiFfYdH8Gipec8Eeeu0xXdbba9frFj0=OqFfea0dXdd9vqai=hGuQ8kuc9pgc9s8qqaq=dirpe0xb9q8qiLsFr0=vr0=vr0dc8meaabaqaciaacaGaaeqabaWaaeGaeaaakeaaimaacqWFxepwaaa@384F@ represents the observed data. The nodes (red circles) represent the *K *values of ℱ
 MathType@MTEF@5@5@+=feaafiart1ev1aaatCvAUfKttLearuWrP9MDH5MBPbIqV92AaeXatLxBI9gBamrtHrhAL1wy0L2yHvtyaeHbnfgDOvwBHrxAJfwnaebbnrfifHhDYfgasaacH8akY=wiFfYdH8Gipec8Eeeu0xXdbba9frFj0=OqFfea0dXdd9vqai=hGuQ8kuc9pgc9s8qqaq=dirpe0xb9q8qiLsFr0=vr0=vr0dc8meaabaqaciaacaGaaeqabaWaaeGaeaaakeaaimaacqWFXeIraaa@3787@ (in this example *K *= 4). Right: Additional layer with random variable ℒ
 MathType@MTEF@5@5@+=feaafiart1ev1aaatCvAUfKttLearuWrP9MDH5MBPbIqV92AaeXatLxBI9gBamrtHrhAL1wy0L2yHvtyaeHbnfgDOvwBHrxAJfwnaebbnrfifHhDYfgasaacH8akY=wiFfYdH8Gipec8Eeeu0xXdbba9frFj0=OqFfea0dXdd9vqai=hGuQ8kuc9pgc9s8qqaq=dirpe0xb9q8qiLsFr0=vr0=vr0dc8meaabaqaciaacaGaaeqabaWaaeGaeaaakeaaimaacqWFsectaaa@376E@, "subcellular localization class", and two conditionally independent observed variables X
 MathType@MTEF@5@5@+=feaafiart1ev1aaatCvAUfKttLearuWrP9MDH5MBPbIqV92AaeXatLxBI9gBamrtHrhAL1wy0L2yHvtyaeHbnfgDOvwBHrxAJfwnaebbnrfifHhDYfgasaacH8akY=wiFfYdH8Gipec8Eeeu0xXdbba9frFj0=OqFfea0dXdd9vqai=hGuQ8kuc9pgc9s8qqaq=dirpe0xb9q8qiLsFr0=vr0=vr0dc8meaabaqaciaacaGaaeqabaWaaeGaeaaakeaaimaacqWFxepwaaa@384F@_1_, X
 MathType@MTEF@5@5@+=feaafiart1ev1aaatCvAUfKttLearuWrP9MDH5MBPbIqV92AaeXatLxBI9gBamrtHrhAL1wy0L2yHvtyaeHbnfgDOvwBHrxAJfwnaebbnrfifHhDYfgasaacH8akY=wiFfYdH8Gipec8Eeeu0xXdbba9frFj0=OqFfea0dXdd9vqai=hGuQ8kuc9pgc9s8qqaq=dirpe0xb9q8qiLsFr0=vr0=vr0dc8meaabaqaciaacaGaaeqabaWaaeGaeaaakeaaimaacqWFxepwaaa@384F@_2_. The different widths of the arrows symbolize different transition probabilities.

We combine both classification schemes by way of a *hidden Markov model *(HMM). This choice was guided by the observation that even the standard *K*-class discriminant analysis model can be viewed as a simple HMM consisting of *K *emitting nodes and a silent *begin *and *end *node, see the left panel of Figure 2. The *K *emitting nodes represent the values of the hidden variable ℱ
 MathType@MTEF@5@5@+=feaafiart1ev1aaatCvAUfKttLearuWrP9MDH5MBPbIqV92AaeXatLxBI9gBamrtHrhAL1wy0L2yHvtyaeHbnfgDOvwBHrxAJfwnaebbnrfifHhDYfgasaacH8akY=wiFfYdH8Gipec8Eeeu0xXdbba9frFj0=OqFfea0dXdd9vqai=hGuQ8kuc9pgc9s8qqaq=dirpe0xb9q8qiLsFr0=vr0=vr0dc8meaabaqaciaacaGaaeqabaWaaeGaeaaakeaaimaacqWFXeIraaa@3787@ ("functional class"). The Gaussian emission probabilities are derived from the classifier. The transition probabilities are estimated by the empirical frequencies of class membership in a training set.

A second classification scheme is included in the automaton by adding another layer with *L *emitting nodes. These additional nodes correspond to a Gaussian mixture model with *L *components that in our case correspond to subcellular localization classes, see the right panel of Figure 2. The emitting nodes in the first layer represent the values of the hidden variable ℒ
 MathType@MTEF@5@5@+=feaafiart1ev1aaatCvAUfKttLearuWrP9MDH5MBPbIqV92AaeXatLxBI9gBamrtHrhAL1wy0L2yHvtyaeHbnfgDOvwBHrxAJfwnaebbnrfifHhDYfgasaacH8akY=wiFfYdH8Gipec8Eeeu0xXdbba9frFj0=OqFfea0dXdd9vqai=hGuQ8kuc9pgc9s8qqaq=dirpe0xb9q8qiLsFr0=vr0=vr0dc8meaabaqaciaacaGaaeqabaWaaeGaeaaakeaaimaacqWFsectaaa@376E@ ("localization"), whereas the nodes in the second layer represent the values of the second hidden variable ℱ
 MathType@MTEF@5@5@+=feaafiart1ev1aaatCvAUfKttLearuWrP9MDH5MBPbIqV92AaeXatLxBI9gBamrtHrhAL1wy0L2yHvtyaeHbnfgDOvwBHrxAJfwnaebbnrfifHhDYfgasaacH8akY=wiFfYdH8Gipec8Eeeu0xXdbba9frFj0=OqFfea0dXdd9vqai=hGuQ8kuc9pgc9s8qqaq=dirpe0xb9q8qiLsFr0=vr0=vr0dc8meaabaqaciaacaGaaeqabaWaaeGaeaaakeaaimaacqWFXeIraaa@3787@ ("functional class"). There are now two "observed data" variables X
 MathType@MTEF@5@5@+=feaafiart1ev1aaatCvAUfKttLearuWrP9MDH5MBPbIqV92AaeXatLxBI9gBamrtHrhAL1wy0L2yHvtyaeHbnfgDOvwBHrxAJfwnaebbnrfifHhDYfgasaacH8akY=wiFfYdH8Gipec8Eeeu0xXdbba9frFj0=OqFfea0dXdd9vqai=hGuQ8kuc9pgc9s8qqaq=dirpe0xb9q8qiLsFr0=vr0=vr0dc8meaabaqaciaacaGaaeqabaWaaeGaeaaakeaaimaacqWFxepwaaa@384F@_1_, X
 MathType@MTEF@5@5@+=feaafiart1ev1aaatCvAUfKttLearuWrP9MDH5MBPbIqV92AaeXatLxBI9gBamrtHrhAL1wy0L2yHvtyaeHbnfgDOvwBHrxAJfwnaebbnrfifHhDYfgasaacH8akY=wiFfYdH8Gipec8Eeeu0xXdbba9frFj0=OqFfea0dXdd9vqai=hGuQ8kuc9pgc9s8qqaq=dirpe0xb9q8qiLsFr0=vr0=vr0dc8meaabaqaciaacaGaaeqabaWaaeGaeaaakeaaimaacqWFxepwaaa@384F@_2 _that are assumed conditionally independent given the states of the two hidden variables. This independence assumption might be justified by the use of sparse kernel selection rules in each layer which typically induce nearly orthogonal feature spaces. The emission probabilities *P*(X
 MathType@MTEF@5@5@+=feaafiart1ev1aaatCvAUfKttLearuWrP9MDH5MBPbIqV92AaeXatLxBI9gBamrtHrhAL1wy0L2yHvtyaeHbnfgDOvwBHrxAJfwnaebbnrfifHhDYfgasaacH8akY=wiFfYdH8Gipec8Eeeu0xXdbba9frFj0=OqFfea0dXdd9vqai=hGuQ8kuc9pgc9s8qqaq=dirpe0xb9q8qiLsFr0=vr0=vr0dc8meaabaqaciaacaGaaeqabaWaaeGaeaaakeaaimaacqWFxepwaaa@384F@_1_|ℒ
 MathType@MTEF@5@5@+=feaafiart1ev1aaatCvAUfKttLearuWrP9MDH5MBPbIqV92AaeXatLxBI9gBamrtHrhAL1wy0L2yHvtyaeHbnfgDOvwBHrxAJfwnaebbnrfifHhDYfgasaacH8akY=wiFfYdH8Gipec8Eeeu0xXdbba9frFj0=OqFfea0dXdd9vqai=hGuQ8kuc9pgc9s8qqaq=dirpe0xb9q8qiLsFr0=vr0=vr0dc8meaabaqaciaacaGaaeqabaWaaeGaeaaakeaaimaacqWFsectaaa@376E@) and *P*(X
 MathType@MTEF@5@5@+=feaafiart1ev1aaatCvAUfKttLearuWrP9MDH5MBPbIqV92AaeXatLxBI9gBamrtHrhAL1wy0L2yHvtyaeHbnfgDOvwBHrxAJfwnaebbnrfifHhDYfgasaacH8akY=wiFfYdH8Gipec8Eeeu0xXdbba9frFj0=OqFfea0dXdd9vqai=hGuQ8kuc9pgc9s8qqaq=dirpe0xb9q8qiLsFr0=vr0=vr0dc8meaabaqaciaacaGaaeqabaWaaeGaeaaakeaaimaacqWFxepwaaa@384F@_2_|ℱ
 MathType@MTEF@5@5@+=feaafiart1ev1aaatCvAUfKttLearuWrP9MDH5MBPbIqV92AaeXatLxBI9gBamrtHrhAL1wy0L2yHvtyaeHbnfgDOvwBHrxAJfwnaebbnrfifHhDYfgasaacH8akY=wiFfYdH8Gipec8Eeeu0xXdbba9frFj0=OqFfea0dXdd9vqai=hGuQ8kuc9pgc9s8qqaq=dirpe0xb9q8qiLsFr0=vr0=vr0dc8meaabaqaciaacaGaaeqabaWaaeGaeaaakeaaimaacqWFXeIraaa@3787@) are learned separately in the two layers. As in the former case, the individual transition probabilities are estimated by empirical frequencies on the training set. For predicting multilabels in the second layer, we compute the posterior probabilities via the forward-backward algorithm. The number of labels to be assigned is again found by thresholding the sum of ordered probabilities, cf. eq. (9). Varying this threshold yields precision-recall curves as depicted in Figure 1.

### Locality due to pairwise kernel classifiers

The method introduced above finds a "global" set of kernels for the full multi-class problem. In some applications, however, it might be desirable to further investigate the discriminative power of kernels in a more class-specific or "local" setting. This can be achieved by an alternative approach to multi-class discriminant analysis based on the *pairwise coupling *scheme in [14]. The main idea is to find a *K*-class discriminant rule (with classes *C*_1_,...,*C*_*K*_) by training all *K*(*K *- 1)/2 possible two-class classifiers and coupling the obtained conditional membership probabilities *r*_*ij *_:= *Prob*(*C*_*i*_|*C*_*i *_or *C*_*j*_) to a consistent *K*-class assignment probability. In other words, we want to find probabilities *p*_1_,...,*p*_*K *_such that the quantities sij:=pip1+pj
 MathType@MTEF@5@5@+=feaafiart1ev1aaatCvAUfKttLearuWrP9MDH5MBPbIqV92AaeXatLxBI9gBaebbnrfifHhDYfgasaacH8akY=wiFfYdH8Gipec8Eeeu0xXdbba9frFj0=OqFfea0dXdd9vqai=hGuQ8kuc9pgc9s8qqaq=dirpe0xb9q8qiLsFr0=vr0=vr0dc8meaabaqaciaacaGaaeqabaqabeGadaaakeaacqWGZbWCdaWgaaWcbaGaemyAaKMaemOAaOgabeaakiabcQda6iabg2da9maalaaabaGaemiCaa3aaSbaaSqaaiabdMgaPbqabaaakeaacqWGWbaCdaWgaaWcbaGaeGymaedabeaakiabgUcaRiabdchaWnaaBaaaleaacqWGQbGAaeqaaaaaaaa@3C78@ are as close as possible to the estimated *r*_*ij*_.

The probabilities *p*_1_,...,*p*_*K *_are finally found be minimizing the KL-divergence between *r*_*ij *_and *s*_*ij*_. This pairwise coupling approach can also be adapted for multiple class labels by way of "fractional" class assignments in the training step. If we find many samples in the training set which belong to both the classes *i *and *j*, multi-label discriminant analysis will effectively shrink the respective class centroids ***μ***_*i *_and ***μ***_*j *_towards their common mean while keeping the covariances constant, a procedure which will favor the co-prediction of classes *i *and *j*. In our experiments, we use this pairwise approach to LDA as a "building block" in the two-layered HMM for simultaneous prediction of subcellular localization and functional class. The advantage of this pairwise method over the "global" approach is that in each of the *K*(*K *- 1)/2 subproblems a task-specific subset of kernels is learned. Concerning the computational workload, the pairwise approach is very similar to the "global" method, since the increase of classifiers to be learned is compensated by the smaller sample size in the individual two-class problems.

## Results and discussion

On the top-level hierarchy, the functional catalog provided by the *MIPS comprehensive yeast genome database *(CYGD) [15] assigns roughly 4000 yeast proteins to several functional classes listed in Table 1. Note that this classification scheme corresponds to an old version of the MIPS functional catalog, whereas the newest version, *funcat 2.0*, further splits some of these 13 classes. To allow a better comparison with previous results reported in [1], however, we still use the older labeling scheme.

### Kernel representation

One of the major advantages of our method is its capability of automatically extracting relevant data sources out of a large collection of kernels presented by the user. The user can, thus, collect as much information sources as possible and let the algorithm decide which to choose. Following this idea, we represent the yeast proteins by previously used kernels that extract information on different levels, like mRNA expression, protein sequences and protein-protein interactions. Moreover, we enrich this set of "basis" kernels by variants thereof resulting from nonlinear feature space mappings. We further investigated additional kernels from several publicly available microarray datasets [16-20].

The "basis" kernels introduced in [1] consist of (i) two kernels which analyze the domain structure: *K*_pfdom _and an enriched variant *K*_pf_exp_; (ii) three diffusion kernels on interaction graphs: *K*_mpi_(protein-protein interactions), *K*_mgi _(genetic interactions), *K*_tap _(co-participation in protein complexes); (iii) two kernels derived from cell cycle gene expression measurements: *K*_exp_d _(binary) and *K*_exp_g _(Gaussian); (iv) a string alignment kernel *K*_SW_. From each of these 8 kernels we derive 3 additional Gaussian RBF variants by computing squared Euclidean distances Dij2
 MathType@MTEF@5@5@+=feaafiart1ev1aaatCvAUfKttLearuWrP9MDH5MBPbIqV92AaeXatLxBI9gBaebbnrfifHhDYfgasaacH8akY=wiFfYdH8Gipec8Eeeu0xXdbba9frFj0=OqFfea0dXdd9vqai=hGuQ8kuc9pgc9s8qqaq=dirpe0xb9q8qiLsFr0=vr0=vr0dc8meaabaqaciaacaGaaeqabaqabeGadaaakeaacqWGebardaqhaaWcbaGaemyAaKMaemOAaOgabaGaeGOmaidaaaaa@3194@ between pairs of objects (*i*, *j*) and deriving new kernels under this nonlinear feature space transform as Kij(l)=exp⁡(−σlDij2)
 MathType@MTEF@5@5@+=feaafiart1ev1aaatCvAUfKttLearuWrP9MDH5MBPbIqV92AaeXatLxBI9gBaebbnrfifHhDYfgasaacH8akY=wiFfYdH8Gipec8Eeeu0xXdbba9frFj0=OqFfea0dXdd9vqai=hGuQ8kuc9pgc9s8qqaq=dirpe0xb9q8qiLsFr0=vr0=vr0dc8meaabaqaciaacaGaaeqabaqabeGadaaakeaacqWGlbWsdaqhaaWcbaGaemyAaKMaemOAaOgabaGaeiikaGIaemiBaWMaeiykaKcaaOGaeyypa0JagiyzauMaeiiEaGNaeiiCaaNaeiikaGIaeyOeI0ccciGae83Wdm3aaSbaaSqaaiabdYgaSbqabaGccqWGebardaqhaaWcbaGaemyAaKMaemOAaOgabaGaeGOmaidaaOGaeiykaKcaaa@43F9@ for the three RBF variants *l *= 1, 2, 3.

### Multilabels

Since a protein can have several functions, each protein comes with a set *Y *of functional class labels. Let |*Y*| denote the cardinality of this set, i.e. the number of classes a certain protein is assigned to. For running *Algorithm 1 *in the methods section below we have to translate the label sets *Y*_*i*_, *i *= 1,...,*n *into membership probabilities which form the entries of the "blurred" *n *× *K *indicator matrix Z˜
 MathType@MTEF@5@5@+=feaafiart1ev1aaatCvAUfKttLearuWrP9MDH5MBPbIqV92AaeXatLxBI9gBaebbnrfifHhDYfgasaacH8akY=wiFfYdH8Gipec8Eeeu0xXdbba9frFj0=OqFfea0dXdd9vqai=hGuQ8kuc9pgc9s8qqaq=dirpe0xb9q8qiLsFr0=vr0=vr0dc8meaabaqaciaacaGaaeqabaqabeGadaaakeaacuWGAbGwgaacaaaa@2DF8@, where *n *is the number of proteins and *K *the number of classes. Since no further information is available, for the *i*-th protein we set Z˜
 MathType@MTEF@5@5@+=feaafiart1ev1aaatCvAUfKttLearuWrP9MDH5MBPbIqV92AaeXatLxBI9gBaebbnrfifHhDYfgasaacH8akY=wiFfYdH8Gipec8Eeeu0xXdbba9frFj0=OqFfea0dXdd9vqai=hGuQ8kuc9pgc9s8qqaq=dirpe0xb9q8qiLsFr0=vr0=vr0dc8meaabaqaciaacaGaaeqabaqabeGadaaakeaacuWGAbGwgaacaaaa@2DF8@_*ik *_= 1/|*Y*_*i*_|, if the *k*-th class is a member of the label set *Y*_*i*_, and zero otherwise. In the following these membership probabilities will also be called "fractional labels".

### Performance evaluation

In [1], all 13 classes were trained separately in a one-against-all manner, where a gene is treated as a member of a certain class whenever it has a positive label for that class (irrespective of other labels!). The performance of these classifiers has been evaluated in terms of *area under the ROC curves *(*auc*). Our method, on the contrary, respects the multilabel structure of the problem by explicitly exploiting co-occurrences of class labels. It uses *fractional labels *Z˜
 MathType@MTEF@5@5@+=feaafiart1ev1aaatCvAUfKttLearuWrP9MDH5MBPbIqV92AaeXatLxBI9gBaebbnrfifHhDYfgasaacH8akY=wiFfYdH8Gipec8Eeeu0xXdbba9frFj0=OqFfea0dXdd9vqai=hGuQ8kuc9pgc9s8qqaq=dirpe0xb9q8qiLsFr0=vr0=vr0dc8meaabaqaciaacaGaaeqabaqabeGadaaakeaacuWGAbGwgaacaaaa@2DF8@_*ik*_, and the output is a probability vector for all classes. Thus, even in the optimal case, our classifier will assign a score of 1/|*Y*_*i*_| to a correct class. Since the test set contains genes with *different *cardinalities of label sets, the classifier scores reside on different scales and it will be impossible to find a common threshold when computing a ROC curve.

To overcome this problem, we use two different measures: for each class *C*_*k*_, *auc*_0_/1 measures the area under the ROC curve only on the subset of genes which either do not belong to class *C*_*k*_, or which exclusively belong to *C*_*k*_. For this subset (≈ 2/3 of the yeast genes) we can directly compute a ROC curve, since there are no scaling problems. The measure *auc*_weighted_, on the other hand, uses all test genes and rescales both the fractional label Z˜
 MathType@MTEF@5@5@+=feaafiart1ev1aaatCvAUfKttLearuWrP9MDH5MBPbIqV92AaeXatLxBI9gBaebbnrfifHhDYfgasaacH8akY=wiFfYdH8Gipec8Eeeu0xXdbba9frFj0=OqFfea0dXdd9vqai=hGuQ8kuc9pgc9s8qqaq=dirpe0xb9q8qiLsFr0=vr0=vr0dc8meaabaqaciaacaGaaeqabaqabeGadaaakeaacuWGAbGwgaacaaaa@2DF8@_*ik *_for class *C*_*k *_and the corresponding probabilistic classifier score *P*(*C*_*k*_|***x***_*i*_) for the *i*-th protein by the label set cardinality, Z˜′ik=Z˜ik⋅|Yi|⇒Z˜′ik∈{0,1}
 MathType@MTEF@5@5@+=feaafiart1ev1aaatCvAUfKttLearuWrP9MDH5MBPbIqV92AaeXatLxBI9gBaebbnrfifHhDYfgasaacH8akY=wiFfYdH8Gipec8Eeeu0xXdbba9frFj0=OqFfea0dXdd9vqai=hGuQ8kuc9pgc9s8qqaq=dirpe0xb9q8qiLsFr0=vr0=vr0dc8meaabaqaciaacaGaaeqabaqabeGadaaakeaacuWGAbGwgaacgaqbamaaBaaaleaacqWGPbqAcqWGRbWAaeqaaOGaeyypa0JafmOwaOLbaGaadaWgaaWcbaGaemyAaKMaem4AaSgabeaakiabgwSixlabcYha8jabdMfaznaaBaaaleaacqWGPbqAaeqaaOGaeiiFaWNaeyO0H4TafmOwaOLbaGGbauaadaWgaaWcbaGaemyAaKMaem4AaSgabeaakiabgIGiolabcUha7jabicdaWiabcYcaSiabigdaXiabc2ha9baa@4C31@.

**Figure 3:**
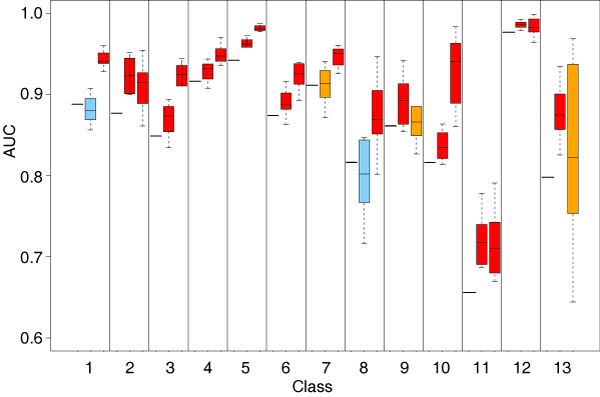
**Performance evaluation**. Performance Evaluation for the 13 functional classes of yeast proteins. From left to right: results from [1], *auc*_weighted _and *auc*_0_/1. Red: significant improvements; orange: improvements of unclear significance; light blue: worse performance (unclear significance).

Figure 3 depicts the results for the enlarged kernel set consisting of the 8 "basis" kernels and 3 additional RBF kernel variants thereof. For each of the 13 classes three performance values (area under ROC curve) are shown: the result reported in [1] (depicted solely as vertical bars since no variance measurements are provided) and the two measures *auc*_weighted _and *auc*_0_/1 for our multilabel approach (represented as box-plots). Each of the latter two significantly outperforms the former in most classes (marked red). The measure *auc*_0_/1 shows an improved median performance in *all *classes (some are probably not significant, marked orange), *auc*_weighted _has worse performance in 2 classes (probably not significant, light blue). A control experiment in which we used only the 8 "basis" kernels yielded a slightly lower performance.

The improvement obtained by using the enlarged kernel set becomes more obvious when computing the *F1-measure *which is the harmonic mean of *precision *and *recall*. The latter are similar to but different from the axes of ROC curves which encode *fallout *and *recall*. Precision is the probability that a predicted category is a true category, whereas *fallout *is the probability that a true absence of a category was labeled a false positive presence. Since the definite absence of a certain protein function can be hardly validated experimentally, the estimated fallout rate will strongly depend on the actual status of experimental coverage. The precision measure, on the other hand, does not so severely suffer from this problem, since the number of present categories might be estimated more reliably even with a small number of carefully designed experiments.

To compute the *F1 *statistics for a given threshold *τ *in eq. (9), we select for each gene the *k *most probable multilabels such that the sum of the *k *largest membership probabilities exceeds *τ*. We then compute *precision *and *recall *up to the rank *k *and combine both to the *F1-measure*. Variation of *τ *yields a complete precision-recall-F1-curve. With the enlarged kernel set the maximum *F1 *value increases significantly, as can be seen in the two leftmost boxplots in Figure 1.

**Figure 4:**
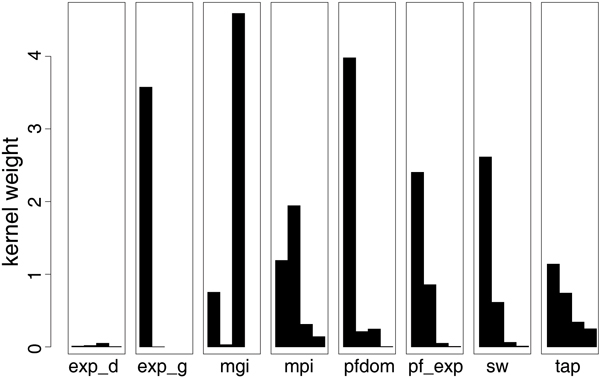
**Learned kernel weights**. Learned kernel weights. Each box contains one of the 8 "basis" kernels and three Gaussian RBF variants (from left to right).

Figure 4 depicts the learned kernel weights. Each box contains 4 bins corresponding to the original kernels from [1] and three Gaussian RBF kernel variants with decreasing kernel width. It is of particular interest that a RBF variant of the genetic interaction kernel *K*_mgi _attains the highest weight, whereas the original diffusion kernel *K*_mgi _on the interaction graph seems to contain almost no discriminative information (consistent with [1] where *K*_mgi _is the least important kernel). The reason for the improved performance of the RBF kernel variant might be the *local *nature of the Gaussian kernel function. A diffusion kernel encodes transition probabilities for a *random walk *model on a graph *G*. In the light of this random walk interpretation, the steep decay of the Gaussian kernel accentuates the local graph structure.

### Functional classes and subcellular localization

**Figure 5:**
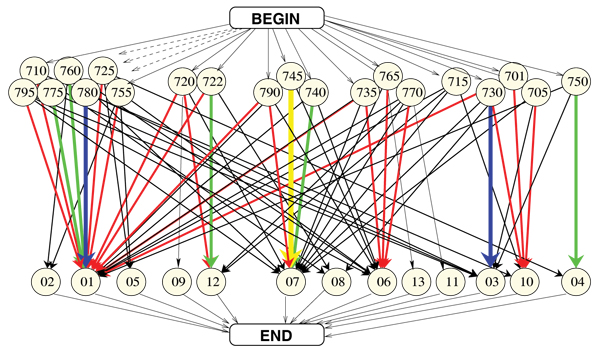
**The hidden Markov model**. The hidden Markov model. Combined graph for subcellular location classes (upper layer) and functional classes (lower layer). Joint predictions of these two entities means finding (multiple) paths through the graph from *begin *to *end*. The nodes in the two layers encode the values of the hidden random variables *location class *and *functional class*, see also Figure 2. The arrows between the nodes encode "transition" probabilities which are estimated by frequency counts on a training set. For highlighting the main structure of this graph, only transition probabilities with *p *> 0.1 are shown. Width and color of the arrows encode these probabilities: > 0.8 yellow, > 0.6 blue, > 0.4 green, > 0.2 red. For instance, the yellow arrow between the nodes "745" and "07" means that more than 80% of the proteins with subcellular localization *transport vesicles *belong to the functional class *cellular transport & transport mechanism*.

For the yeast genome MIPS CYGD provides a classification scheme with respect to subcellular localization of the proteins, see Table 2. We combine both the functional and the localization-based scheme by way of the *hidden Markov model *(HMM) described in the methods section below. The corresponding automaton model is depicted in Figure 5. The first layer contains 20 emitting nodes corresponding to the top-level localization classes in Table 2. The transition probabilities are estimated from a training set by counting the occurrences of paths in the model. In order to highlight the essential graph structure, only the transitions with probability above 0.1 are shown. Note that several localization nodes have dominant transitions to only one or two functional nodes, see e.g. node "750" (*nucleus*) which has a strong prior for class "04" (*transcription*), the pair "730" (*cytoskeleton*) and "03" (*cell cycle & DNA processing*), or "745" (*transport vesicles*) which strongly votes for functional class "07" (*cellular transport & transport mechanism*).

In order to evaluate the possible advantages of the combined localization-function classifier, we again conducted a cross-validation experiment in which both sets of labels were predicted. According to the results summarized in the left panel of Figure 1, the inclusion of the prediction step for the subcellular localization of a protein indeed improves the prediction of its function.

### Towards a "local" model: pairwise kernel weights

While all previous models find a common set of kernels for all classes, the pairwise coupling approach described in the methods section couples pairwise classifiers which find individual kernel weights that are optimal for the "local" problem of separating only two classes. These pairwise classifiers can again learn class correlations induced by objects with multiple labels.

The rightmost boxplot in the left panel of Figure 1 shows that this "local" model significantly improves the predictive power of the HMM-based classifier. The right panel depicts the evolution of precision, recall and F1 under variation of the threshold *τ *in eq. (9).

**Figure 6:**
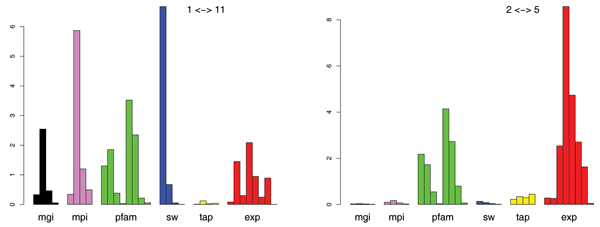
**Kernel weights for the pairwise model**. Kernel weights for the pairwise model. Left: separating classes *metabolism *and *control of cellular organization*. Right: classes *energy *and *protein synthesis*. The kernels are arranged in groups according to their origin: genetic interaction (mgi), prot.-prot. interaction (mpi), domain structure (pfam) string alignments (SW), protein complexes (tap) and gene expression (exp). The 8 gene expression RBF kernels represent the data in [16-20] and three RBF kernel variants of the data in [21] (left to right).

Figure 6 shows two examples of the learned kernel weights in the 78 individual two-class models for the 13 functional classes which nicely demonstrate the adaptiveness of the pairwise approach: while for the separation of the classes *metabolism *and *control of cellular organization *the combination of Smith-Waterman sequence alignment kernels ("SW") and protein interaction kernels ("mpi") have a main role, the separation of classes *energy *and *protein synthesis *is dominated by gene expression information ("exp") and protein domain structure ("pfam"). A closer analysis of all 78 classifiers shows some general trends, for instance the importance of gene expression kernels whenever one of the classes *energy *or *protein synthesis *is involved.

## Conclusion

While kernel-based classifiers have been successfully applied to a variety of prediction tasks, their main drawback is the lacking interpretability of the decision functions. One attempt to overcome this shortcoming is to find a weighted combination of multiple kernels, each of which represents a different type of measurement. The idea is that sparse kernel combinations allow the user to identify the relevant influence factors for a given task. In this work, the problem of learning such sparse kernel combinations has been addressed by reformulating classification as an indicator regression problem using adaptive ridge penalties. While the standard adaptive ridge model presented in [11] selects individual input features, our extensions concerning *weight sharing *and *kernelization *lead to a nonlinear model that finds sparse combinations of *kernel matrices*. A probabilistic treatment of multiple labels allows us to apply the classifier to tasks in which the input objects can belong to more than one category. The effect of multilabels can be intuitively understood as shrinking the centroids of classes which share many multilabeled objects towards their average centroid, thus favoring co-prediction of these classes.

The method has been applied to the problem of predicting the function of yeast proteins which defines a classical multilabel setting. From the experiments we conclude that our model compares favorably to the approach in [1]. Two aspects seem to be of particular importance: on the modeling side, our approach directly exploits the multilabel structure of the problem, rather than ignoring class correlations.

Concerning the computational aspects, the efficiency of the method allows us to easily enlarge the set of kernels: as long as one single matrix can be hold in the main memory, the algorithm is highly efficient. For yeast proteins, the use of additional kernels has e.g. lead to the insight that genetic interactions are highly discriminative for functional predictions.

Aiming at a still higher classification rate and at more detailed information about the relevance of kernels, we have introduced two further modifications: extending the multilabel classifier to a two-layer *hidden Markov model *(HMM) allows us to combine two different labeling schemes. Multilabel prediction in the HMM naturally translates to reconstructing multiple paths through a graph. It could be shown that the prediction of the subcellular localization of a protein in the first layer helps to identify its functional class in the second layer, the reason for this improvement being the strong correlation of nodes in both layers. Localization in the *transport vesicles*, for instance, gives a strong prior for having a role in the functional class *cellular transport & transport mechanism*.

The second modification concerns the transition from a single "global" prediction model to several "local" models which focus on the separation of *pairs of classes *only. The estimated pairwise membership probabilities are coupled to a consistent set of assignment probabilities over all classes. Using the pairwise classifiers as "building blocks" in the HMM offers the advantage of increased adaptiveness, since the kernel weighting can focus on the individual requirements for separating one class from another. This approach leads not only to a significantly increased classification performance, but it also gives a much more detailed picture on the importance of different data sources for predicting protein function.

## Authors' contributions

Both authors developed methods, performed comparisons of methods, and wrote the manuscript.

## Figures and Tables

**Table 1:** Functional classes from MIPS CYGD

01	metabolism	08	cell rescue, defense
02	energy	09	interaction w/cell.envt.
03	cell cycle & DNA processing	10	cell fate
04	transcription	11	control of cell. org.
05	protein synthesis	12	transport facilitation
06	protein fate	13	others
07	cellular transp. & transp. mech.		

**Table 2:** Top-level hierarchy of subcellular localization classes from CYGD

701	extracellular	745	transport vesicles
705	bud	750	nucleus
710	cell wall	755	mitochondria
715	cell periphery	760	peroxisome
720	plasma membrane	765	endosome
722	integral membrane/endo membranes	770	vacuole
725	cytoplasm	775	microsomes
730	cytoskeleton	780	lipid particles
735	ER	790	punctate composite
740	golgi	795	ambiguous
